# Co-regulated gene expression by oestrogen receptor α and liver receptor homolog-1 is a feature of the oestrogen response in breast cancer cells

**DOI:** 10.1093/nar/gkt827

**Published:** 2013-09-17

**Authors:** Chun-Fui Lai, Koen D. Flach, Xanthippi Alexi, Stephen P. Fox, Silvia Ottaviani, Paul T.R. Thiruchelvam, Fiona J. Kyle, Ross S. Thomas, Rosalind Launchbury, Hui Hua, Holly B. Callaghan, Jason S. Carroll, R. Charles Coombes, Wilbert Zwart, Laki Buluwela, Simak Ali

**Affiliations:** ^1^Department of Surgery and Cancer, Imperial College London, London W12 0NN, UK, ^2^Department of Molecular Pathology, The Netherlands Cancer Institute, 1066 CX Amsterdam, Netherlands and ^3^Cancer Research UK, Cambridge Research Institute, Li Ka Shing Centre, Cambridge CB2 0RE, UK

## Abstract

Oestrogen receptor α (ERα) is a nuclear receptor that is the driving transcription factor expressed in the majority of breast cancers. Recent studies have demonstrated that the liver receptor homolog-1 (LRH-1), another nuclear receptor, regulates breast cancer cell proliferation and promotes motility and invasion. To determine the mechanisms of LRH-1 action in breast cancer, we performed gene expression microarray analysis following RNA interference for LRH-1. Interestingly, gene ontology (GO) category enrichment analysis of LRH-1–regulated genes identified oestrogen-responsive genes as the most highly enriched GO categories. Remarkably, chromatin immunoprecipitation coupled to massively parallel sequencing (ChIP-seq) to identify genomic targets of LRH-1 showed LRH-1 binding at many ERα binding sites. Analysis of select binding sites confirmed regulation of ERα−regulated genes by LRH-1 through binding to oestrogen response elements, as exemplified by the TFF1/pS2 gene. Finally, LRH-1 overexpression stimulated ERα recruitment, while LRH-1 knockdown reduced ERα recruitment to ERα binding sites. Taken together, our findings establish a key role for LRH-1 in the regulation of ERα target genes in breast cancer cells and identify a mechanism in which co-operative binding of LRH-1 and ERα at oestrogen response elements controls the expression of oestrogen-responsive genes.

## INTRODUCTION

Oestrogens play diverse roles in the body, most notably in the development and maintenance of female and male reproductive systems and secondary sexual characteristics (1). Oestrogens are also implicated in the physiology of the brain, bone and the cardiovascular system, as evidenced by the increased risk of cardiovascular disease and osteoporosis following the decline in oestrogen levels during menopause (1–3). Oestrogens also play a central role in promoting breast cancer growth (4), as well as being implicated in uterine and ovarian cancers (5,6). Two closely related members of the nuclear receptor (NR) superfamily of transcription factors, oestrogen receptor α (ERα) and ß (ERß) mediate oestrogen actions (7,8). The majority (70–80%) of breast cancers express ERα, and this transcription factor is believed to drive cancer cell proliferation. Therefore, ERα activity is inhibited in breast cancer patients with endocrine therapies using anti-oestrogens, such as tamoxifen and fulvestrant or by inhibiting oestrogen biosynthesis either by using aromatase inhibitors in postmenopausal women or with lutenising hormone releasing hormone (LHRH) agonists in premenopausal women. These therapies are well-tolerated and have been a major factor in the improvement in patient survival seen in recent years. However, up to 50% of patients with ERα-positive disease that would require endocrine therapies do not respond, while many responders eventually relapse, with few treatment options being available following the development of resistance (4,9). Hence, a better understanding of the mechanisms of ERα action would aid patient stratification and identify new therapeutic targets.

Ligand binding to ERα promotes recruitment to cis-regulatory regions by direct binding to DNA at oestrogen response elements (EREs) in target genes, or indirectly through interaction with other transcription factors such as AP1 and Sp1 (10,11) and consequent activation or repression of gene expression. Chromatin immunoprecipitation (ChIP) studies have shown that oestrogen addition initiates cycles of ERα association and dissociation from EREs, with concomitant cycles of transcriptional co-regulator recruitment leading to chromatin remodelling and histone modification and cyclical recruitment of the RNA polymerase II (PolII) machinery and subsequent transcription initiation (12–15). These cycles of co-regulator and PolII recruitment are accompanied by cycles of reversal and re-establishment of many, although not all, induced chromatin modifications.

While it is incontrovertible that ERα drives the growth response in the majority of breast tumours, there is mounting evidence that ERα does not act on its own and that other transcription factors are essential for ERα action in breast cancer. Gene expression profiling and genomic approaches for genome-wide identification of ERα binding regions, such as ChIP-chip and ChIP-seq, have allowed the identification of direct ERα targets in breast cancer cells (16–19) and in tumours (20). These studies have also highlighted the importance of other transcription factors in the ERα response, such as FoxA1 (also known as HNF3α). FoxA1 expression is associated with ERα positivity in breast cancer and FoxA1 is one of the minimal set of genes that define ERα-positive luminal cancer (21). FoxA1, which has been proposed to facilitate binding of other transcription factors to DNA through its action in promoting chromatin accessibility (22), is frequently present at regions of ERα binding in the absence of oestrogen and appears to be a key determinant of ERα binding following oestrogen addition (16,23,24). GATA proteins also act as ‘pioneer factors’, promoting transcription factor recruitment (22), and GATA3 guides ERα chromatin interactions and its expression is strongly associated with ERα-positive luminal A breast cancer subtype (25). Thus, FoxA1, GATA3, as well as the transcription factors AP-2γ, TLE1 and PBX1, act as pioneer factors for ERα DNA binding by promoting chromatin accessibility and long-range chromatin interactions (26,27). Interestingly, some of these pioneer factors are themselves ERα-regulated genes, indicating a feed-forward mechanism that can act to reinforce oestrogenic signals in breast cancer cells.

A role in ERα signalling for other NRs has recently been highlighted by the finding that retinoic acid receptor-α, an oestrogen-regulated gene in breast cancer (28,29), localizes to ERα binding sites to modulate the expression of oestrogen-regulated genes (30,31), providing crosstalk between oestrogen and retinoid signalling in breast cancer cells. Moreover, androgen receptor (AR) expression is strongly associated with ERα positivity and AR inhibits expression of oestrogen-responsive genes in ERα-positive breast cancer cells (32). Indeed, a small subset of breast tumours, termed the ‘molecular apocrine’ subtype, which are ERα-negative but express AR (33), typically express genes normally expressed in ERα-positive breast cancer (34). Recent expression microarray and AR ChIP-seq data generated for a cell line characteristic of molecular apocrine breast cancer, MDA-MB-453 (ERα-/PR-/AR+), showed that AR activates transcription of many typical ERα target genes through AR recruitment to sites that are normally bound by ERα in luminal MCF-7 cells (35). These findings indicate another mode of crosstalk between ERα and other NRs mediated by co-operative and/or antagonistic interactions.

Liver receptor homolog-1 (LRH-1) (NR5A2) is expressed in developing and adult tissues of endodermal origin, including liver, pancreas, intestine and the ovary (36,37). Functionally, LRH-1 has been implicated in the regulation of bile acid and cholesterol homeostasis (36,37) and the regulation of inflammatory responses in the liver and gut (38). LRH-1 is also important for steroid hormone biosynthesis in the ovary (39), but also extra-ovarian tissues, including preadipocytes, where LRH-1 regulates aromatase expression (40,41). LRH-1 is also critical in development. Disruption of the LRH-1 gene in mice is embryonic lethal at day E6.5–7.5 (42), and LRH-1 regulates expression of the key pluripotency factor Oct4 in the developing embryo epiblast (43). Indeed, LRH-1 can replace Oct4 in the reprogramming of mouse somatic cells to induced pluripotent stem cells and induces expression of Nanog, a transcription factor important for maintaining pluripotency in undifferentiated embryonic stem cells (44,45).

LRH-1 has also been implicated in cancer. Mice heterozygous for an adenomatosis polyposis coli (APC) mutation and an LRH-1 inactivating mutation developed fewer intestinal tumours than mice harbouring the APC mutation only, and LRH-1 heterozygous mice developed fewer azoxymethane-induced aberrant crypt foci (46). LRH-1 expression is elevated in pancreatic cancer and promotes pancreatic cancer cell growth through stimulation of cyclin D1, cyclin E1 and c-Myc (47), while genome-wide association studies implicate mutations in the LRH-1 gene in pancreatic ductal adenocarcinoma (48). Aromatase is an LRH-1 target gene, which catalyses the conversion of androgens (primarily androstenedione and testosterone) to oestrogens. Aromatase expression and activity is low in breast cancer cells; rather there is an increase in aromatase expression/activity in tumour-bearing breast stroma compared with normal breast stroma, leading to the proposal that LRH-1 may aid breast cancer progression in postmenopausal women by promoting local oestrogen biosynthesis (40,41,49).

Although the real importance of local oestrogen production for breast cancer remains unclear (50,51), recent work demonstrates that LRH-1 is also expressed in breast cancer cells where its expression is ERα-regulated (52,53). In breast carcinoma, LRH-1 expression is associated with ERα positivity (54). Functional studies have shown that LRH-1 plays a direct role in regulating breast cancer cell proliferation and promotes breast cancer cell motility and invasion (52,53,55). Based on these findings, we have identified LRH-1–regulated genes using gene expression microarray profiling and ChIP-seq to map LRH-1 binding events in proliferating breast cancer cells. We show that LRH-1 is an important regulator of oestrogen-responsive gene expression that shares many binding sites with ERα. Importantly, at shared sites, LRH-1 promotes ERα recruitment and vice versa, ERα stimulates LRH-1 recruitment, thus providing evidence for a novel mode of NR co-operativity in breast cancer cells.

## MATERIALS AND METHODS

### Cell culture

MCF-7 and COS-1 cells, obtained from American Type Culture Collection (LGC Standards, UK), were routinely cultured in Dulbecco’s modified Eagle’s medium (DMEM) containing 10% foetal calf serum (FCS). For oestrogen depletion experiments, the cells were transferred to DMEM lacking phenol red and containing 5% dextran-coated charcoal-stripped FCS (DSS) for 72 h. 17ß-estradiol (oestrogen) was added to a final concentration of 10 nM, and ICI182 780 (fulvestrant) was added to a final concentration of 100 nM. The synthetic LRH-1 agonist, compound 5A was added to a final concentration of 30 µM (53,56).

### Plasmids

The Renilla luciferase reporter gene was RLTK (Promega, UK). The LRH-1 expression plasmid pCI-LRH-1 and the LRH-1 firefly luciferase reporter gene SF-1-luc were gifts from Dr Donald McDonnell (57). HA-tagged LRH-1 (pCI-HA3-LRH-1) was generated by insertion of 3xHA coding sequence from pMXB-3HA-mNR5A2 (44) into pCI-LRH-1 to generate HA-tagged human LRH-1 variant 4 (53). The ERα expression plasmid has been described previously (58). The pS2-luc and pS2-ΔERE-luc reporter genes were kindly provided by Dr Vincent Giguere (59). pS2-luc mutants were generated by site-directed mutagenesis using the Quickchange kit from Stratagene, UK.

### Reporter gene assays

For transient transfection, COS-1 cells were seeded in 24-well plates in DMEM lacking phenol red and supplemented with 5% DSS. Following seeding for 24 h, cells were transfected using FuGENE HD (Promega, UK), with 100 ng of luciferase reporter genes, 10 ng of ERα and 50 ng of LRH-1. Oestrogen (10 nM) or compound 5A (30 µM) were added as indicated. Luciferase activities were determined after a further 24 h, using the Dual-Glo Luciferase Assay kit (Promega, UK). RLTK was transfected to control for transfection efficiency, so firefly luciferase activities were calculated relative to the Renilla luciferase (RLTK) activities.

### siRNA transfections

Cells were transfected with double-stranded RNA oligonucleotides using the Lipofectamine RNAiMax reverse transfection method (Invitrogen, UK), according to manufacturer’s protocols. ON-TARGETplus SMARTpool for LRH-1 (LU-003430, Thermofisher), or individual siRNAs from the SMARTpool, were used as indicated in figure legends. siLuc control (D-002050; Thermofisher) or Control siRNA (1027251; Qiagen) were transfected as negative controls. All siRNA experiments used the double-stranded RNA oligonucleotides at a final concentration of 80 nM.

### Western blotting

Cells were cultured and whole-cell lysates were prepared as described previously (60). Antibodies used for western blotting are detailed in Supplementary Materials.

### Quantitative reverse transcriptase-polymerase chain reaction

Total RNA was collected and real-time reverse transcriptase-polymerase chain reaction (RT-PCR) was performed as previously described (61). Real-time RT-PCR was carried out using Taqman Gene Expression Assays (Applied Biosystems, UK). Assay details can be found in the Supplementary Materials.

### Electrophoretic mobility shift assay

HA-LRH-1 and ERα proteins, made using the TNT coupled transcription/translation system (Promega), were used for electrophoretic mobility shift assay (EMSA), as described previously (62), with the exception that the oligonucleotides were labelled at the 5′ end with DY782, allowing detection of complexes using a LiCoR Odyssey Infrared imaging system.

### Chromatin Immunoprecipitations

ChIPs were performed as described previously (63). For each ChIP, 10 µg of antibody (detailed in Supplementary Information) and 100 µl of Dynalbeads Protein A (10002D; Invitrogen) were used. Primers for real-time PCR are also provided in Supplementary Materials.

### ChIPs and Solexa sequencing

ChIP DNA was amplified as described (63). Sequences were generated by the Illumina Hiseq 2000 genome analyser (using 50 bp reads), and aligned to the Human Reference Genome (assembly hg19, February 2009). Enriched regions of the genome were identified by comparing the ChIP samples with an input sample using the MACS peak caller (64) version 1.3.7.1. All ChIP-seq analyses were performed in duplicate, where only the peaks shared by both replicates were considered. The numbers of reads obtained, the percentage of reads aligned and the number of peaks called are detailed in Supplementary Figure S1.

### Motif analysis, heatmaps and genomic distributions of binding events

ChIP-seq data snapshots were generated using the Integrative Genome Viewer IGV 2.2 (www.broadinstitute.org/igv/) and the UCSC genome browser (http://genome.ucsc.edu). Motif analyses were performed through the Cistrome (cistrome.org), applying the SeqPos motif tool (65). The genomic distributions of binding sites were analysed using the *cis*-regulatory element annotation system (CEAS) (66). The genes closest to the binding site on both strands were analysed. If the binding region is within a gene, CEAS software indicates whether it is in a 5′ untranslated region (5′ UTR), a 3′ untranslated region (3′ UTR), a coding exon or an intron. Promoter is defined as 3 kb upstream from RefSeq 5′ start. If a binding site is >3 kb away from the RefSeq transcription start site, it is considered distal intergenic. For integration with gene expression data, binding events were considered proximal when identified in a gene body or within 20 kb upstream of the transcription start site. Heatmaps were generated using Seqminer, using default settings (67).

### Gene expression microarray analysis

MCF-7 cells were transfected with LRH-1 siRNA #2, #3 or with a non-targeting siRNA (siControl; D-001210-10, Thermofisher). RNA was extracted 72 h later using the QIAGEN RNeasy Kit (Qiagen Ltd., UK). Following assessment of RNA integrity, four independent biological replicates for each siRNA treatment were used for microarray analysis. The analysis was performed using HumanHT-12 v 3.0 Expression BeadChiP (Illumina). The BeadChiP image data were preprocessed using GenomeStudio (Illumina). The expression data were then log_2_ transformed and quantile-normalized using Partek Genomics Suite. Gene ontology (GO) analyses were performed using the database for annotation, visualization and integrated discovery (DAVID) (http://david.abcc.ncifcrf.gov) (68). The microarray data have been deposited with the NCBI Gene Expression Omnibus (GEO) (http://ncbi.nlm.nih.gov/geo/) under accession number GSE47803. The ChIP-seq data are available under accession number GSE49390.

## RESULTS

### LRH-1 regulates the expression of oestrogen-responsive genes in breast cancer cells

**Figure 1.**
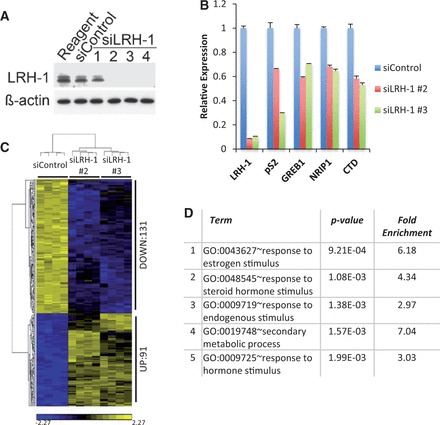
Expression profiling shows that LRH-1 regulates the expression of oestrogen-regulated genes. (**A**) Western blot analysis of MCF-7 cell extracts prepared following transfection with control siRNA or four independent siRNAs for LRH-1. (**B**) Quantitative RT-PCR analysis was performed using three independent RNA preparations made from siControl– or siLRH-1–transfected MCF-7 cells. Error bars represent standard errors of the mean (SEM). (**C**) Gene expression profiling was carried out using four independent replicates of MCF-7 cells transfected with siControl, siLRH-1 #2 or siLRH-1 #3. Shown are significant differentially expressed genes at *P* < 0.01 corrected for false discovery rate (FDR), together with a fold change >1.5 and <−1.5. (**D**) GO category enrichments of the genes differentially regulated by siLRH-1 are shown.

Recent studies have shown that LRH-1 regulates breast cancer cell proliferation and motility (52,53,55). To identify genes that mediate LRH-1 action in breast cancer cells, global gene expression profiling was performed following LRH-1 silencing using siRNAs. RNA was prepared from MCF-7 cells transfected with two independent LRH-1 siRNAs that gave >90% LRH-1 knockdown (Figure 1A and B). Hierarchical cluster analysis identified 222 genes the expression of which was significantly altered (*P* < 0.01, fold change >1.5) with both siRNAs, when compared with the control siRNA (siControl) (Figure 1C). GO analysis showed that the most highly enriched GO terms for these genes were ‘response to oestrogen stimulus’ and ‘response to hormone stimulus’ (Figure 1D), implicating LRH-1 in the regulation of oestrogen-regulated genes in breast cancer cells.

**Figure 2.**
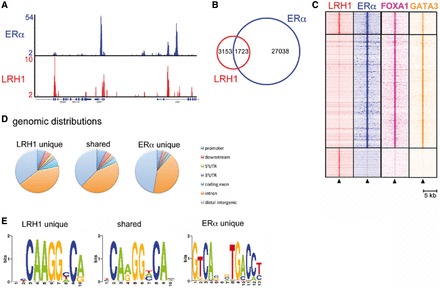
LRH-1 associates with ERα binding regions in chromatin. (**A**) ChIP-seq was carried out using HA and ERα antibodies for HA-LRH-1–transfected MCF-7 cells. Genome browser snapshot of ChIP-seq samples for ERα and HA-LRH-1 on proliferating cells. Y bar shows tag count. (**B**) Venn diagram of LRH-1 and ERα binding events. (**C**) Shown is a heatmap, with a window of 5 kb around the binding sites, depicting all shared and unique binding events for LRH-1 and ERα vertically aligned. Additionally, the binding events of the FOXA1 (24) and GATA3 (25) ChIP-sequencing data sets are shown. (**D**) The genomic distribution of shared and unique binding events. (**E**) *De novo* motif analysis of the shared and unique ERα and LRH-1 binding events.

To determine if LRH-1 directly regulates the expression of oestrogen-responsive genes, ChIP-seq in MCF-7 cells was carried out using antibodies for LRH-1. However, none of the antibodies commercially available provided sufficient enrichment for peak calling to identify LRH-1 binding regions (data not shown). A recent study utilized ectopic expression of epitope-tagged LRH-1 to identify LRH-1–regulated genes in mouse embryonic stem cells (44). Using a similar strategy, we performed ChIP-seq following transfection of MCF-7 cells with HA-tagged LRH-1 (as exemplified in Figure 2A). Aligned reads were acquired and following stringent cut-offs using two independent replicates, peak calling identified 4876 LRH-1 binding sites, of which 1723 (35%) were shared with ERα (Figure 2B). The shared and unique binding patterns were not dictated by different thresholds in peak calling algorithms, as shown by heatmap analyses (Figure 2C and Supplementary Figure S1). Previous publications have demonstrated that the vast majority of ERα binding events map to enhancers and introns, with few sites mapping to promoter proximal regions. The majority of LRH-1 and ERα binding events, both unique and shared, were similarly located within introns and regions distal to gene promoters (Figure 2D).

The majority of NRs bind as dimers to sequence motifs arranged as two copies of a hexameric motif, 5′-AGGYCR-3′, organized as direct or inverted repeats, as exemplified by ERα and the ERE (7). A C-terminal extension to the canonical zinc binding motifs in NR DNA binding domains mediates binding of several NRs to DNA response elements 3 bp longer than the standard hexameric sequences. SF1 and LRH-1 bind as monomers to a sequence having the consensus 5′-YCAAGGYCR-3′, where YCA is the 5′ extension to the AGGYCR sequence to which most NR bind (36,69). Analysis of the LRH-1 binding sites for enriched DNA sequences identified a motif similar to this consensus LRH-1 binding element, with the sequence 5′-SYCARGGYCA-3′ (Figure 2E and Supplementary Figure S2). A motif consistent with the ERE consensus site was the top hit in the ERα unique sites, while the shared LRH-1/ERα sites were enriched in the LRH-1 motif. Motif analyses using a candidate scanning approach identified NR binding motifs, with the top hits for the LRH-1 unique sites being the SF1 and LRH-1 binding motifs. For the shared events, SF1/LRH-1 and ESR1 sites were enriched, while the ESR1 site was the top hit for the ERα unique sites. This analysis also highlighted enrichment for TFAP2C (AP2γ), FOXA1, GATA3 and AP-1 (Fos, Jun) sites in the analysis of the ERα unique sites (Supplementary Figure S3). This enrichment is consistent with previous global binding site studies for ERα (26,70). TFAP2C and FOS/JUN sites were also enriched in the LRH-1 unique and LRH-1/ERα shared sites (Supplementary Figures S4 and S5). Interestingly, FOXA1 and GATA3, being essential components of the ERα transcription complex and its activity (24,25), were found present at the LRH-1/ERα shared and ERα unique sites. Both FOXA1 and GATA3 were absent from the regions only bound by LRH-1, suggesting that these factors may not be important for LRH-1 binding, at least in the case of the LRH-1 unique sites.

### LRH-1 binds to EREs in regulatory regions of a subset of oestrogen-regulated genes

**Figure 3.**
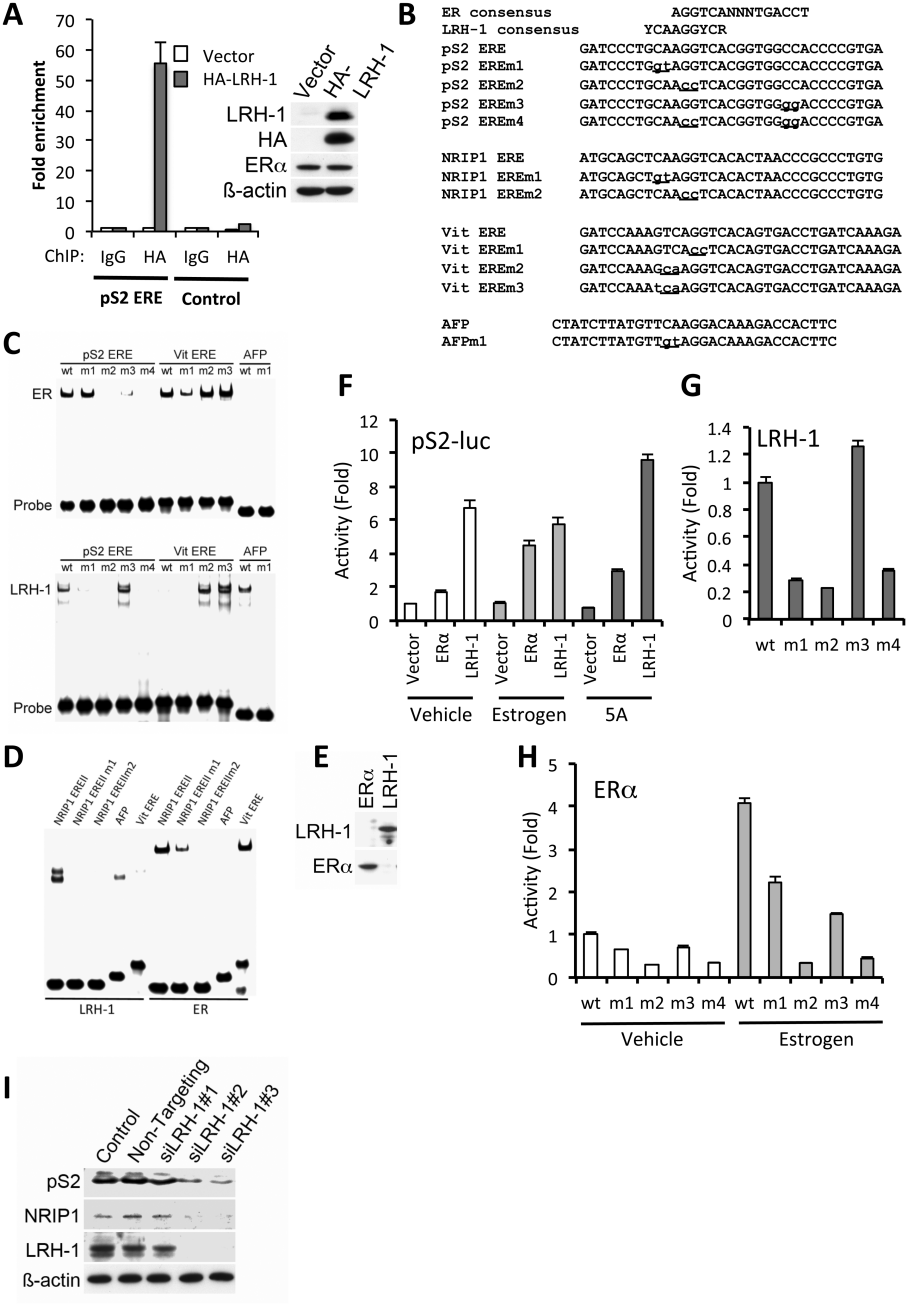
LRH-1 regulates pS2 gene expression through binding to the pS2 ERE. (**A**) ChIP was carried out using IgG (control) or HA antibodies, following transfection of MCF-7 cells with empty vector or HA-LRH-1, followed by real-time PCR using primers for the pS2 ERE region, or with primers amplifying a region upstream of the pS2 ERE (control), as described (14). For each PCR, enrichment is shown relative to the vector control, as mean values for three replicates; error bars represent SEM. Western blotting of HA-LRH-1–transfected MCF-7 cell extracts is also shown. (**B**) Sequences for consensus ERα and LRH-1 binding sites are shown above the sequence of the pS2 ERE. The NRIP1 intronic ERE, chicken vitellogenin ERE and the LRH-1 binding sites in the mouse AFP gene. Mutations generated in these sequences are shown in lower case and are underlined. (**C–E**) EMSA was carried out using oligonucleotides having the sequences shown in (B). LRH-1 and ERα were produced using coupled *in vitro* transcription/translation (IVTT). Western blotting of IVTT products for LRH-1 and ERα is shown in (E). (**F**) COS-1 cells were transfected with pS2-luc, together with ERα and LRH-1, as shown. Reporter gene activities were corrected for transfection efficiency by normalizing to Renilla luciferase activity and are shown relative to reporter gene activity for the vehicle-treated vector control. Shown are the mean pS2-luc activities from three independent transfections; errors bars = SEM. (**G** and **H**) COS-1 cells were transfected with LRH-1 (G) or ERα (H), together with pS2-luc or mutant reporters, as shown. Following normalization for the transfection control (renilla), activities are shown relative to the activities obtained for pS2-luc.

To explore the potential link between LRH-1 and ERα in regulation of common target genes, we focused on the archetypal ERα target gene, pS2, for mechanistic investigations (Supplementary Figure S6A). LRH-1 recruitment to the ERα binding region was confirmed by ChIP (Figure 3A). EMSA demonstrated LRH-1 binding to the pS2 ERE and confirmed the expected sequence requirements for LRH-1 binding (Figure 3B, C and E). Mutation within the 5′-AGGTCA motif prevented LRH-1 and ERα binding, whereas mutation in the 3′ motif prevented ERα binding, but did not influence LRH-1 binding. Similar results were obtained for the ERE motif within the shared LRH-1 and ERα binding region in the NRIP1 gene, another well-studied ERα-regulated gene (Figure 3D and Supplementary Figure S6A). The importance of the 5′ sequences for LRH-1 binding was shown by mutation of the chicken vitellogenin gene ERE to introduce the 5′ extension. These mutations allowed LRH-1 binding to the vitellogenin ERE.

LRH-1 stimulated a reporter gene encoding 1006 bp of the pS2 gene promoter, containing the ERE (59). This ERE sequence is required for activation of pS2 expression by LRH-1 because deletion of the ERE sequence (pS2ΔERE-luc) largely abrogated reporter gene activation by LRH-1 (Figure 3F and Supplementary Figure S6B). Substitutions in the 5′ extension, the 5′ hexameric sequence, but not in the 3′ hexameric motif, prevented LRH-1 activation of the pS2 reporter gene (Figure 3G). Together, these findings demonstrate that LRH-1 can bind to the pS2 gene promoter proximal ERE, to stimulate pS2 gene expression.

LRH-1 knockdown in MCF-7 cells resulted in a reduction in pS2 and NRIP1 expression for two independent siRNAs (Figures 1B and 3I). The expression of AGR3, PDZK1 and RET, genes to which LRH-1 is recruited at ERα binding regions (Supplementary Figure S7), was similarly reduced on LRH-1 knockdown (Supplementary Figure S8). Together, these results show that LRH-1 can be recruited to ERα binding regions, and that binding to select EREs allows LRH-1 regulation of ERα target genes.

### Synergistic recruitment between LRH-1 and ERα to EREs at ERα binding regions

**Figure 4.**
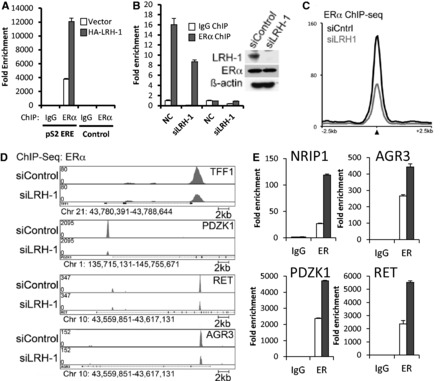
LRH-1 promotes ERα binding to the ERα binding sites. (**A**) ERα ChIP was carried out using chromatin prepared for the ChIP shown in Figure 3A. Enrichment is shown relative to the IgG control as mean values for three replicates. (**B**) ERα ChIP was performed following MCF-7 treatment with siControl (NC) or siLRH-1 (*n* = 3). Western blotting for LRH-1 and ERα is also shown. (**C**) Average signal intensity of ERα binding events is shown for MCF-7 cells treated with siControl or siLRH-1. (**D**) Genome browser snapshots of ChIP-seq samples for ERα from siControl– or siLRH-1–treated MCF-7 cells are shown. Y bar shows tag count. (**E**) ERα ChIP for ERα binding regions is shown for vector (clear bars) and LRH-1–transfected (grey bars) MCF-7 cells (*n* = 3, error bars = SEM).

As shown above, LRH-1 knockdown gave a marked reduction in expression of pS2, NRIP1 and other genes for which LRH-1 binding to ERα binding regions was observed by ChIP-seq, demonstrating that LRH-1, like ERα, promotes expression of these genes. This raises the possibility that ERα and LRH-1 co-operatively regulate expression of ERα target genes. Indeed, ectopic expression of LRH-1 in MCF-7 cells stimulated ERα recruitment to the pS2 promoter (Figure 4A). Conversely, siLRH-1 treatment reduced ERα recruitment to the pS2 promoter (Figure 4B). To extend this finding, ChIP-seq for ERα following siLRH-1 treatment was carried out to determine the importance of LRH-1 for ERα binding to ERα binding sites. LRH-1 knockdown resulted in a reduction by ∼50% in the intensity of ERα binding events (Figure 4C and Supplementary Figure S9A). The reduction in ERα binding exemplified for pS2 (TFF1), PDZK1, RET and AGR3 (Figure 4D) was confirmed by ChIP-QPCR (Supplementary Figure S9B–E). Moreover, ectopic expression of LRH-1 stimulated ERα binding at these regions (Figure 4A and E; Supplementary Figure S10). The ELOVL2 gene serves as an example of an ERα binding site to which LRH-1 is not recruited (Supplementary Figure S10). In this case, LRH-1 silencing did not influence ERα recruitment (Supplementary Figure S9F).

**Figure 5.**
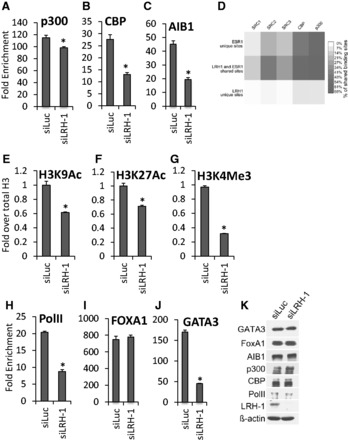
LRH-1 is required for co-activator loading and histone modification. MCF-7 cells were transfected with LRH-1 siRNA. ChIP was performed using antibodies for ERα cofactors (**A–C**, **I** and **J**), histone H3 acetylation and methylation marks (**E–G**) or PolII (**H**), followed by real-time PCR of the pS2 ERE, as shown. Enrichment is shown relative to the IgG control (*n* = 3). The acetylated and methylated H3 ChIP was first normalized to total H3, then to the IgG control. Errors bars = SEM, *P < 0.05. (**D**) Heatmap of ERα co-activator binding [data from (71)], showing the percentage of overlapping SRC1, SRC2, SRC3, CBP and p300 binding events at LRH-1 and ERα unique and shared sites. (**K**) Western blotting of the lysates in parts (A–C, E–J), is shown.

To determine if LRH-1 functions to promote or maintain cofactor recruitment, we performed ChIP for p300 and CREB Binding Protein (CBP), well-known ERα co-activators (12) following LRH-1 silencing. There was a significant decrease in p300 and CBP binding at all loci (Figure 5A and B). Binding of the AIB1 co-activator was also decreased in the absence of LRH-1 (Figure 5C). These data illustrate that the LRH-1 and ERα shared sites are also occupied by typical ERα co-activators. Mining publically available data sets for SRC1, SRC2, SRC3 (AIB1), p300 and CBP (71) illustrated that this was indeed the case, and cofactor recruitment was found enriched at LRH-1/ERα sites on a genome-wide level. There was, however, no appreciable difference in p300 binding. This suggests that co-operativity between LRH-1 and ERα promotes co-activator recruitment at co-regulated genes. The changes in cofactor binding were reflected in changes in chromatin structure following LRH-1 silencing, as demonstrated by reduction in levels of histone marks associated with gene expression, namely acetylation of histone H3 (Figure 5E–G). Also reduced on LRH-1 silencing were levels of H3 lysine 4 trimethylation (H3K4Me3), a marker of transcriptional activity (72) at the pS2 promoter (Figure 5F), as well as PolII recruitment (Figure 5H). These findings, together with the reduction in mRNA levels of these genes (Supplementary Figure S8), are further indicative of a requirement for LRH-1 for the transcription of these oestrogen-responsive genes. Note that there was no change in levels of these proteins with LRH-1 knockdown (Figure 5K).

Interestingly, LRH-1 knockdown did not affect FOXA1 binding to ERα binding regions (Figure 5I). This may be a reflection of the described presence of FOXA1 before ERα recruitment and its requirement for ERα recruitment to chromatin (16,23,24). The importance of FOXA1 has also been demonstrated for other NRs, e.g. AR (35), and it might also be required for LRH-1 recruitment. However, binding of GATA3, which is also critical for ERα recruitment, was significantly reduced following LRH-1 silencing (Figure 5J), identifying another mechanism by which LRH-1 regulates ERα recruitment. There was no reduction in FOXA1 or GATA3 protein levels on LRH-1 knockdown (Figure 5K).

**Figure 6.**
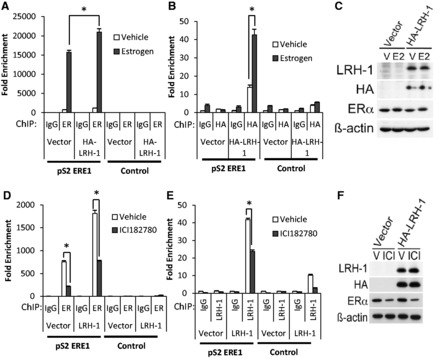
Modulation of ERα activity regulates LRH-1 recruitment to ERα binding regions. MCF-7 cells transfected with HA-LRH-1 were treated with oestrogen for 45 min (**A–C**) or with ICI182 780 for 24 h (**D–F**). ChIP for ERα (A and D) or LRH-1 (B and E) was performed. The mean enrichment relative to the IgG control for three independent experiments is shown. Errors bars = SEM, *P < 0.05. (C and F) Western blotting of the lysates is shown.

The above results demonstrate a requirement for LRH-1 for the transcription of oestrogen-responsive genes in breast cancer cells. However, treatment of MCF-7 cells with oestrogen stimulated LRH-1 binding to these sites (Figure 6A–C and Supplementary Figure S11). Moreover, treatment of MCF-7 cells with ICI182 780, an anti-oestrogen that results in ERα degradation, reduced LRH-1 binding (Figure 6D–F and Supplementary Figure S12). Note that levels of ectopically expressed LRH-1 are unaffected by oestrogen or ICI182 780 treatment. Together, these results demonstrate that as LRH-1 stimulates ERα binding, ERα reciprocally promotes LRH-1 recruitment.

## DISCUSSION

LRH-1 is a NR transcription factor that plays important roles in development, reproduction and metabolism. Its role in reverse cholesterol transport and bile acid homeostasis has been especially well studied, and it has been shown that LRH-1 acts as a potentiation factor for the LXR oxysterol receptors through binding to sites proximal to LXR binding sites to induce expression of key genes involved in these processes, including CYP7A1, CYP8B1, CETP, SREB-1c and FAS (36,73–77). The bile acid receptor FXR is also implicated in crosstalk with LRH-1 and ChIP-seq for FXR and LRH-1 reveals that almost a quarter of hepatic LRH-1 binding sites are located in close proximity to FXR binding sites, suggesting that co-operativity between LRH-1 and FXR is important for the regulation of metabolic genes in the liver (78,79).

LRH-1 is a direct ERα target gene (52,53), its expression correlates with ERα in breast tumours (54) and it promotes breast cancer proliferation and invasion (55). We now show that LRH-1 is an important regulator of ERα target genes because LRH-1 siRNA resulted in reduced expression of oestrogen-responsive genes. Moreover, LRH-1 ChIP-seq analysis shows that a substantial proportion of LRH-1 binding sites map to ERα binding sites, with enrichment of the LRH-1 binding motif being evident at these sites. This indicates that LRH-1 functions in breast cancer cells not only by mediating oestrogen action at non-ERα target genes following stimulation of its expression by ERα (which may be viewed as a classic mechanism by which a signal transduction pathway response may be amplified) but also by potentiating ERα action at many genes that are direct targets of ERα. The co-operativity between LRH-1 and ERα was further illustrated by the substantial overlap in binding sites between the ERα unique binding sites and ERα/LRH-1 shared sites with FOXA1 and GATA3, both well-established parts of the ERα transcription complex.

Of particular note was our observation of overlapping LRH-1 and ERα binding sites, to which binding of both factors was confirmed. This is in contrast to the metabolic genes regulated by LRH-1/LXR and LRH-1/FXR, where binding at proximal but non-overlapping sites has been described (73–79). Analysis of one of these overlapping binding sites, at the pS2 promoter ERE shows that LRH-1 binds to the 5′ hexamer of this ERE, the sequence around this hexamer being consistent with the extended sequence to which LRH-1 binds. This finding is consistent with a previous report that showed LRH-1 binding to the pS2 and GREB1 EREs (80). We further show here that LRH-1 stimulated pS2 expression and LRH-1 knockdown showed that it is required for pS2 expression. Interestingly, LRH-1 overexpression promoted ERα recruitment, while LRH-1 knockdown by siRNA inhibited ERα binding to the pS2 ERE, indicative of a requirement of LRH-1 for ERα binding. Similar promotion of ERα recruitment by LRH-1 was observed for the NRIP1 intronic ERE, as well as ERα binding regions associated with other oestrogen-responsive genes. ERα ChIP-seq following LRH-1 silencing also demonstrated a reduction in ERα binding, consistent with an important role for LRH-1 in promoting ERα binding. Further investigation of the LRH-1 ChIP-seq data also showed detectable LRH-1 signal at additional ERα binding sites, excluded by the peak call algorithm, due to threshold stringency (Supplementary Figure S13). This suggests that LRH-1 is recruited, albeit weakly, to a considerable proportion of ERα binding sites, further highlighting the importance of LRH-1 for ERα action in breast cancer cells.

Our data are indicative of co-occupancy of the two receptors at a proportion of ERα binding sites. However, we failed to reChIP ERα following ChIP for LRH-1, or vice versa. Nor were we able to obtain evidence for co-binding of ERα and LRH-1 in EMSA, using the pS2 ERE oligonucleotides. Immunoprecipitation of ERα did not co-immunoprecipitate LRH-1 in MCF-7 cells, nor did we detect an interaction in co-immunoprecipitation experiments in COS-1 cells following LRH-1 and ERα overexpression (data not shown). Together, these results indicate that the co-operativity between LRH-1 and ERα does not involve co-occupancy of binding sites by these receptors. This raises the possibility that these receptors bind the pS2 ERE through a sequential recruitment mode of action, so-called ‘assisted loading’, recently described for binding of the glucocorticoid receptor (GR) and a mutant oestrogen receptor that can bind to a GR response element (81). This study demonstrated that binding of one NR does not reduce steady state binding of another NR; rather binding of one NR can facilitate subsequent binding of a second NR by promoting chromatin accessibility, leading to steady state levels of several NRs at the same response element. In agreement with this possibility, LRH-1 silencing reduced levels of GATA3 and co-activator recruitment, histone modifications associated with active chromatin as well as PolII binding. These results suggest that LRH-1 promotes cofactor recruitment and chromatin changes that facilitate ERα recruitment. Additionally, ChIP following the addition of oestrogen promoted LRH-1 recruitment, while ERα downregulation with ICI182 780 treatment inhibited LRH-1 binding, suggestive of a mechanism involving cyclical binding of ERα and LRH-1 to the regulatory regions of oestrogen-responsive genes. These findings show that ERα and LRH-1 co-regulate many oestrogen target genes and highlight LRH-1 as an important mediator of the oestrogen response in breast cancer cells.

The importance of LRH-1 for the expression of oestrogen-responsive genes described here identifies it as a putative drug target in breast cancer. Given that AR has been shown to regulate the expression of ERα target genes in ERα-negative breast cancer (35), it will be interesting to determine the potential importance of LRH-1 in the regulation of ERα target genes in endocrine resistant breast cancer and/or in ERα-negative breast cancer and consequently its therapeutic potential in breast cancer subtypes currently lacking targeted therapies.

## SUPPLEMENTARY DATA

Supplementary Data are available at NAR Online.

## FUNDING

Cancer Research UK [C37/A12011, C37/A9335 to R.C.C., L.B. and S.A.]; Breast Cancer Campaign [2007MayPR17 to S.A.]; Cancer Research UK China Fellows scheme (to H.H.); Medical Research Council Doctoral Training award (to H.C.); Dutch Cancer Society KWF fellowship and a Netherlands Organisation for Scientific Research NWO Veni grant (to W.Z.). Funding for open access charge: Imperial College London.

*Conflict of interest statement*. None declared.

## Supplementary Material

Supplementary Data
