# Supplementary material for: Co-regulated gene expression by oestrogen receptor α and liver receptor homolog-1 is a feature of the oestrogen response in breast cancer cells
Source: Nucleic Acids Res. 2013 Sep 17;41(22):10228–40. doi: 10.1093/nar/gkt827 (PMC3905875; doi:10.1093/nar/gkt827)
Supplement: Supplementary Data [file supp_41_22_10228__index.html]

Co-regulated gene expression by oestrogen receptor α and liver receptor homolog-1 is a feature of the oestrogen response in breast cancer cells — Co-regulated gene expression by oestrogen receptor α and liver receptor homolog-1 is a feature of the oestrogen response in breast cancer cells — Supplementary Data 

# Co-regulated gene expression by oestrogen receptor α and liver receptor homolog-1 is a feature of the oestrogen response in breast cancer cells

## Supplementary Data

files

**Files in this Data Supplement:**

- Supplementary Data - pdf file
