# Supplementary material for: Co-regulated gene expression by oestrogen receptor α and liver receptor homolog-1 is a feature of the oestrogen response in breast cancer cells
Source: Nucleic Acids Res. 2013 Sep 17;41(22):10228–40. doi: 10.1093/nar/gkt827 (PMC3905875; doi:10.1093/nar/gkt827)
Supplement: Supplementary Data [file supp_gkt827_nar-01711-x-2013-File008.pdf]

	ChIP against	Total tag count	Number of tags after filtering	Percentage of tags after filtering	Number of called peaks
**Rep1 siControl**	*ERα*	25070841	22585368	90.1	46913
**Rep1 siLRH1**	*ERα*	20877689	19740866	94.6	31748
**Rep2 siControl**	*ERα*	24363924	22466024	92.2	31934
**Rep2 siLRH1**	*ERα*	19206354	17816547	92.8	35637
**Rep1 Control**	*ERα*	24936940	24285478	97.4	32808
**Rep1 HA-LRH1**	*ERα*	25169008	24737818	98.3	27178
**Rep2 Control**	*ERα*	22829742	19127352	83.8	85660
**Rep2 HA-LRH1**	*ERα*	24729358	22481763	90.9	76557
**Rep1 Control**	*HA-LRH1*	25466376	25415163	99.8	556
**Rep1 HA-LRH1**	*HA-LRH1*	24517660	24411661	99.6	10270
**Rep2 Control**	*HA-LRH1*	12619838	12202611	96.7	186
**Rep2 HA-LRH1**	*HA-LRH1*	6718673	6585960	98	12229

Supplementary Figure 1. 0etails of ChIP-sequencing runs. Included are the Immunoprecipitated proteins (ChIP against), the total number of tag counts, the number and relative percentage of tags left after filtering and the number of called peaks.

LRH-1 Unique Sites	Consensus	Occurences	Cut-off	Z-score	p-value
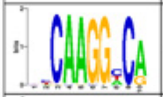	SYGAAGGHCA	3379	-0.206	-38.54	>1.0E-30
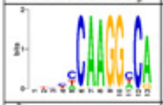	5C1AGG1C1	3298	2.397	-37.50	>1.0E-30
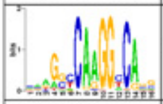	6CA1GG1C3	3331	1.737	-37.49	>1.0E-30
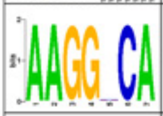	AAGGCCA	3046	3.212	-33.58	>1.0E-30
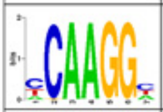	YCAAGGH	2994	6.024	-31.92	>1.0E-30
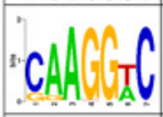	CAAGGWC	2230	5.97	-30.25	>1.0E-30
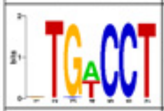	STGWCCT	2177	6.739	-20.82	>1.0E-30
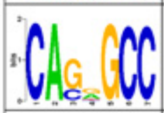	CASRGCC	3362	1.554	-13.10	>1.0E-30
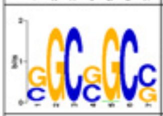	SGCSGCS	3240	-2.079	-12.71	>1.0E-30
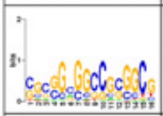	4G2G1C3GC1	3381	-5.546	-11.99	>1.0E-30
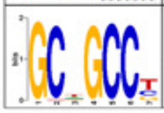	GCWGCCY	3381	-3.156	-11.12	5.2E-29

LRH-1/ESR1 Shared S	Consensus	Occurences	Cut-off	Z-score	p-value
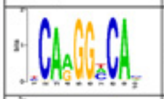	YCARGGTCAS	1019	6.349	-16.57	>1.0E-30
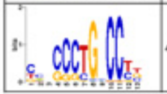	4CC1G1CC2	1606	2.138	-7.99	6.9E-16

ESR1 Unique Sites	Consensus	Occurences	Cut-off	Z-score	p-value
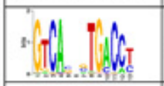	G1CA4G1C2	3091	3.78	-40.00	>1.0E-30
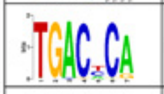	TGACHCA	2912	3.711	-20.70	>1.0E-30
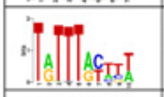	TRTTTRYTTW	3584	4.575	-13.37	>1.0E-30
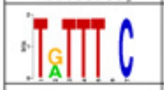	TRTTTDC	2825	7.02	-13.00	>1.0E-30
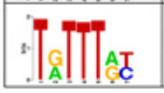	TRTTTRY	4608	2.301	-10.46	6.3E-26

Supplementary Figure 2. Motif analysis of the shared and unique ERα and LRH-1 binding sites. Binding site analysis was carried out using the Cistrome (www.cistrome.org), applying the SeqPos motif tool (He, H. H. et al (2010) Nucleosome dynamics define transcriptional enhancers. Nature Genet., 42: 343-347.

	ID	Transcription Factor	Consensus	Occurences	Z-score	p-value
ESR1 Unique Sites	**MA0112**	**ESR1**	**2G1CA3T2C7**	**3001**	**4.8548**	**>1E-30**
	MA0258	ESR2	4GTCA7C2	3260	4.7987	>1E-30
	M01268	NR1H4	RTGACCY	3942	5.4682	>1E-30
	hPDI020	ZNF655	TSACCT	3195	6.5372	>1E-30
	MA0066	PPARG	4GG6T2CC3	1722	3.5524	>1E-30
	M01589	ESRRB	SSSTGACCTTGR	4658	2.3732	>1E-30
	M01282	PPARA	TGACCTY	4232	4.9149	>1E-30
	**MC00052**	**TFAP2C**	**2CC3GG1C2**	**3133**	**3.7364**	**>1E-30**
	MC00051	TFAP2A	3CC3GG1C1	3154	3.7389	>1E-30
	UP00048	RARA	4TGACC7	4377	3.0031	>1E-30
	UP00009	NR2F2	4TGACC7	4848	2.0197	>1E-30
	MA0160	NR4A2	GTGACCTT	3699	5.3612	>1E-30
	**MA0148**	**FOXA1**	**TGTTTRCWYWG**	**3338**	**5.2597**	**>1E-30**
	M00515	PPARG::RXRA	5GG2A3T3C5	1408	-0.12	>1E-30
	M01722	RORB	TGACCYA	1336	6.5782	>1E-30
	UP00010	TCFAP2B	2GCC4GG3	2236	4.686	>1E-30
	EN0033	RXRA	TGACCTTTGC	4991	2.7853	>1E-30
	UP00079	ESRRA	7ACCTT5	4637	2.4315	>1E-30
	MC00023	FOXA2	2T1TTT1C4	2408	6.0651	>1E-30
	M01267	FOSL1	TGAGTCAK	1688	6.9437	>1E-30
	MC00022	FOSL2	TGASTCA	1385	5.5391	>1E-30
	MC00044	SMARCC1	VTGASTCAYC	1109	7.031	>1E-30
	M01248	NR0B1	7G1CCTT7	3371	4.8115	>1E-30
	EN0110	BCL11A	TGASTMA	2179	5.6492	>1E-30
	EN0123	SMARCA4	VTGASTCABC	1067	7.2472	>1E-30
	EN0339	PAX5	SHGTSAC	3829	4.4447	>1E-30
	MC00029	JUND	GATGASTCAY	983	6.3783	>1E-30
	UP00028	TCFAP2E	3SCC3GG4	3748	2.7464	>1E-30
	UP00087	Tcfap2c	1T2CC3GG4	4130	2.5946	>1E-30
	**MC00019**	**FOS**	**RTGASTCAYM**	**1028**	**7.1689**	**>1E-30**
	**MC00028**	**JUN**	**GRTGASTCAY**	**964**	**7.2634**	**>1E-30**
	**EN0245**	**GATA3**	**GATDWYATCW**	**937**	**7.1105**	**>1E-30**
	M01138	RORA	TGACCYANYWW	4149	3.4512	>1E-30
	UP00005	Tcfap2a	1T2CC3GG4	3362	3.7697	>1E-30
	M01217	NR4A1	YTGACCTTBS	4727	3.157	>1E-30
	hPDI182	ZNF71	TGACCYYT	4879	2.9129	>1E-30
	MC00005	RXRG	GGTCAAAGGTCA	4840	-2.3163	>1E-30
	M01198	NR1H2::RXRA	1G1T1A1AG4	4675	-3.5555	>1E-30
	M00490	BACH2	SRTGAGTCAYS	3074	4.6755	>1E-30
	M01153	NR1I2::RXRA	TGAMCTBY	3626	5.3709	>1E-30
	M01132	SF1	TGRCCTTGR	4853	3.595	>1E-30
	M00495	BACH1	4TGAGT6	810	6.6998	>1E-30
	M01725	NR2C2,Nr2c2	CSTGACCTYTS	3653	4.1886	>1E-30
	EN0271	SMARCB1	STGASTCABC	1283	6.7567	>1E-30
	MA0035	GATA1	RSWGATAAGRR	2566	5.8195	>1E-30
	MC00030	JUND	ATGACKY	3997	3.9566	>1E-30
	EN0365	SIRT6	RSWGATAAGR	2642	5.3143	>1E-30
	EN0272	SMARCB1	WGATAAG	2676	5.6808	>1E-30
	EN0265	HMGN3	YYTTATCWSY	1793	6.3597	>1E-30
	EN0418	NR2C2	1GG2A2GG3	4819	3.6591	>1E-30
	EN0232	FOXP2	GTAAACA	4211	4.7141	>1E-30
	MA0067	PAX2	VMRTGACW	3304	5.2508	>1E-30
	hPDI075	THRA	GTKRCC	3082	6.277	>1E-30
	M00037	NFE2	TGCTGASTCAY	4936	0.5958	>1E-30
	EN0125	CCNT2	YCTTATCWSY	1972	6.2512	>1E-30
	M00821	NFE2L2	6AGTCA2	3303	4.353	>1E-30
	M00348	GATA2	AGWGATAASA	2685	5.3694	>1E-30
	MC00043	SMARCA4	GATAAGR	2794	5.762	>1E-30
	M00041	ATF2::JUN	TRACGTCA	3209	3.0744	>1E-30
	M00983	MAF	TGCTGAGTCAK	1724	6.2115	>1E-30
	hPDI045	ZNF695	WRACCYT	2863	6.2288	>1E-30
	EN0002	ATF3	6CGTCA3	4978	0.1905	>1E-30
	M00526	NR6A1	7TTGA1C5	3422	0.354	>1E-30
	MA0031	FOXD1	GTAAACAW	1756	6.5411	>1E-30
	UP00066	HNF4A	5TGACC7	4907	1.6721	1.31E-29
	MC00011	ATF3	SYCACGTGAC	4835	2.1847	3.26E-29
	M00179	ATF2	RSTKACGTCASB	4911	-0.354	2.62E-28
	EN0372	SP1	3CA1AG2C2	3319	4.8968	4.48E-26
	MC00010	FOXJ3	RTAAACAA	2655	5.755	4.25E-25
	hPDI175	ID2	GAYGWC	2474	5.0716	4.82E-25

Supplementary Figure 3. Motif analysis of ERα unique sites was carried out using a candidate screening, applying the SeqPos motif tool in Cistrome (cistrome.org). Highlighted are motifs described in previous studies as being enriched at ERα binding regions.

	ID	Transcription Factor	Consensus	Occurences	Z-score	p-value
LRH-1/ESR1 Shared Sites	**M01132**	**SF1**	**YCAAGGYCA**	**1721**	**-22.9915**	**>1E-30**
	M01589	ESRRB	SSSTGACCTTGR	1769	-22.1235	>1E-30
	M01217	NR4A1	YTGACCTTBS	1499	-20.2321	>1E-30
	**M01142**	**NR5A2**	**CKRRCCTTGVAC**	**1645**	**-19.293**	**>1E-30**
	M01282	PPARA	RAGGTCA	1599	-16.8451	>1E-30
	MA0160	NR4A2	AAGGTCAC	1553	-16.6283	>1E-30
	UP00079	ESRRA	7ACCTT5	1734	-15.8737	>1E-30
	hPDI020	ZNF655	AGGTSA	1360	-15.6413	>1E-30
	M01268	NR1H4	RGGTCAY	1544	-14.8109	>1E-30
	M01248	NR0B1	7G1CCTT7	1260	-14.6607	>1E-30
	M00526	NR6A1	7TTGA1C5	1528	-13.1625	>1E-30
	**MA0112**	**ESR1**	**7G2A3TG1C2**	**1102**	**-12.0105**	**>1E-30**
	EN0033	RXRA	GCAAAGGTCA	1772	-11.9367	>1E-30
	MA0258	ESR2	2G7TGAC4	1095	-11.218	1.66E-29
	MC00022	FOSL2	TGASTCA	568	-10.9503	3.31E-28
	MC00051	TFAP2A	3CC3GG1C1	1766	-10.569	2.08E-26
	M01267	FOSL1	TGAGTCAK	538	-10.4929	4.66E-26
	**MC00028**	**JUN**	**GRTGASTCAY**	**407**	**-9.9587**	**1.16E-23**
	**MC00052**	**TFAP2C**	**2CC3GG1C2**	**1778**	**-9.8612**	**3.07E-23**
	MC00044	SMARCC1	GRTGASTCAB	438	-9.8568	3.20E-23

Supplementary Figure 4. Motif analysis of LRH-1 and ERα shared sites was carried out using a candidate screening, applying the SeqPos motif tool in Cistrome (cistrome.org). Highlighted are motifs described in previous studies as being enriched at ERα binding regions.

	ID	Transcription Factor	Consensus	Occurences	Z-score	p-value
LRH-1 Unique Sites	**M01132**	**SF1**	**TGRCCTTGR**	**2803**	**-36.4651**	**>1E-30**
	**M01142**	**NR5A2**	**CKRRCCTTGVAC**	**3117**	**-32.2572**	**>1E-30**
	M01217	NR4A1	YTGACCTTBS	2792	-31.7033	>1E-30
	M01589	ESRRB	SSSTGACCTTGR	3041	-29.7035	>1E-30
	M01282	PPARA	RAGGTCA	2938	-24.529	>1E-30
	UP00079	ESRRA	7ACCTT5	2602	-22.0194	>1E-30
	M01248	NR0B1	7G1CCTT7	2943	-21.5178	>1E-30
	MA0160	NR4A2	AAGGTCAC	2770	-19.8636	>1E-30
	M00526	NR6A1	7TTGA1C5	2768	-16.4063	>1E-30
	hPDI020	ZNF655	AGGTSA	1868	-14.9425	>1E-30
	MC00051	TFAP2A	3CC3GG1C1	3131	-14.6209	>1E-30
	M01268	NR1H4	RGGTCAY	2559	-14.4778	>1E-30
	MA0146	ZFX	8GGCCT1	3143	-13.9387	>1E-30
	**MC00052**	**TFAP2C**	**2CC3GG1C2**	**3045**	**-13.8407**	**>1E-30**
	EN0033	RXRA	TGACCTTTGC	2013	-13.6291	>1E-30
	MC00040	REST	3GGA1AG7	3009	-11.9872	>1E-30
	**MC00028**	**JUN**	**GRTGASTCAY**	**645**	**-11.6578**	**>1E-30**
	MC00022	FOSL2	TGASTCA	832	-11.6153	>1E-30
	MC00044	SMARCC1	GRTGASTCAB	593	-11.5513	>1E-30
	hPDI045	ZNF695	ARGGTYW	1999	-11.3735	2.83E-30
	M01253	CNOT3	GGCCGCGSSS	3149	-11.2807	8.17E-30
	EN0271	SMARCB1	STGASTCABC	497	-10.9191	4.67E-28
	M01267	FOSL1	TGAGTCAK	788	-10.8526	9.69E-28
	EN0123	SMARCA4	GVTGASTCAB	741	-10.7673	2.46E-27
	**MC00019**	**FOS**	**KRTGASTCAY**	**587**	**-10.6961**	**5.31E-27**

Supplementary Figure 5. Motif analysis of LRH-1 unique sites was carried out using a candidate screening, applying the SeqPos motif tool in Cistrome (cistrome.org). Highlighted are motifs described in previous studies as being enriched at ERα binding regions.

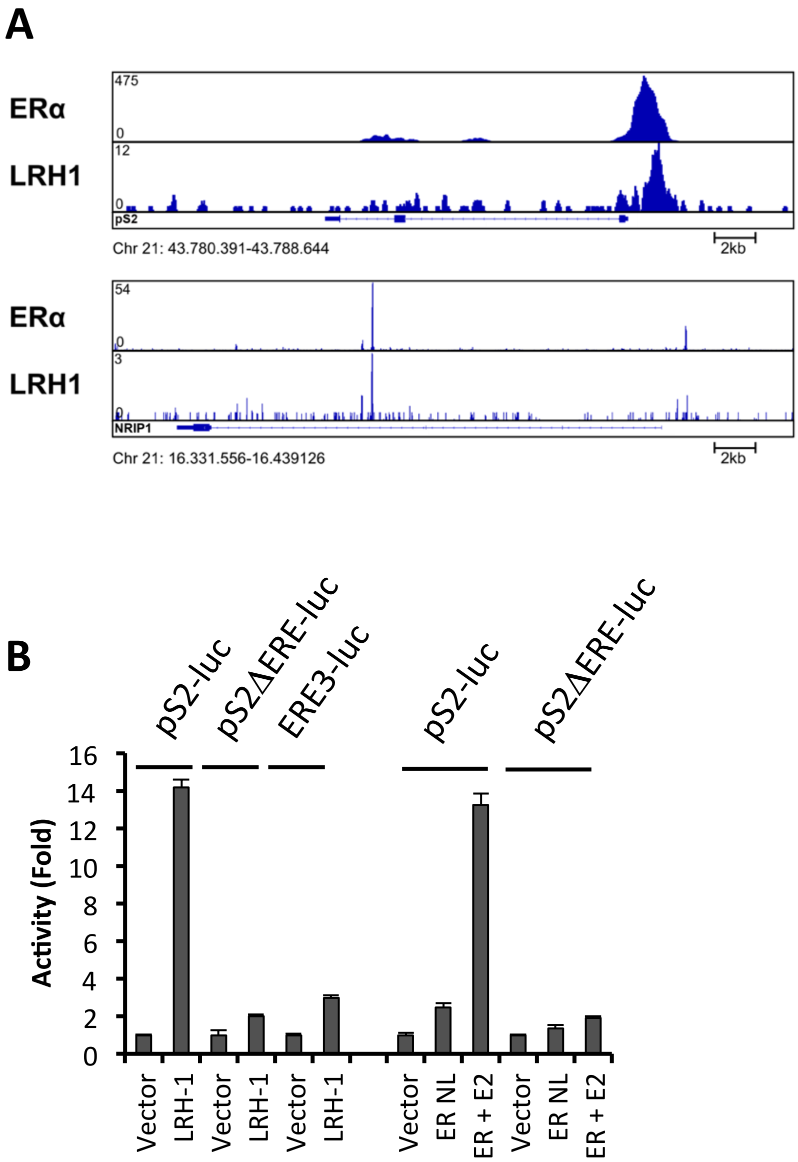

Supplementary Figure 6. (A) Genome browser snapshots of ChIP-seq samples for ERα and LRH-1 on proliferating cells. (B) COS-1 cells, cultured in were transfected with pS2-luc, pS2-luc in which the ERE has been deleted (pS2ΔERE-luc) or ERE3-luc, together with ERα or LRH-1 as indicated. 17ß-estradiol (E2; 10 nM) was added where indicated, an equal volume of vehicle being added to the no ligand (NL) controls. Reporter gene activities are shown relative to the vector controls (n=3; error bars = SEM).

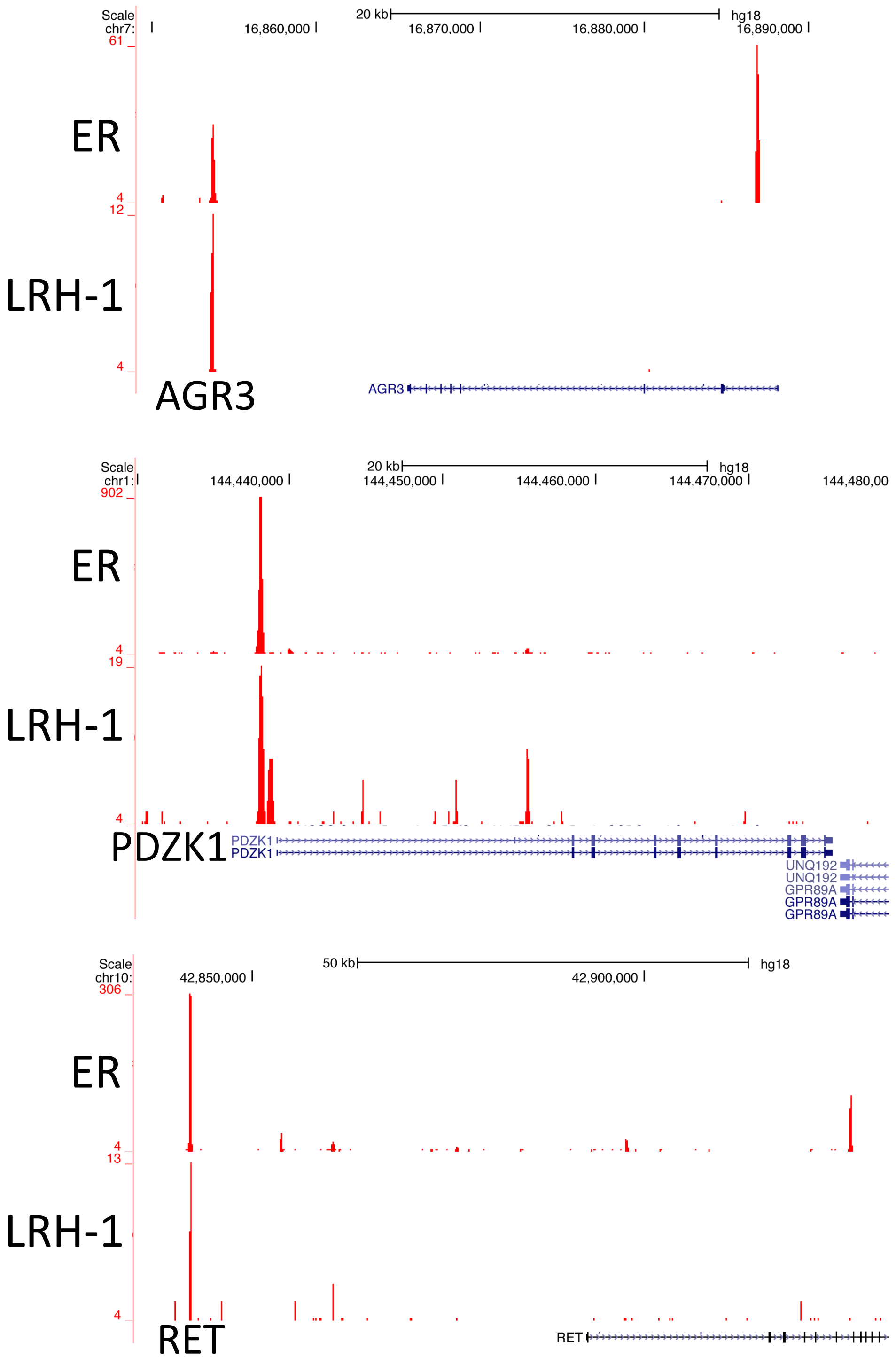

Supplementary Figure 7. Genome browser snapshots of ChIP-seq samples for ERα and LRH-1 on proliferating cells.

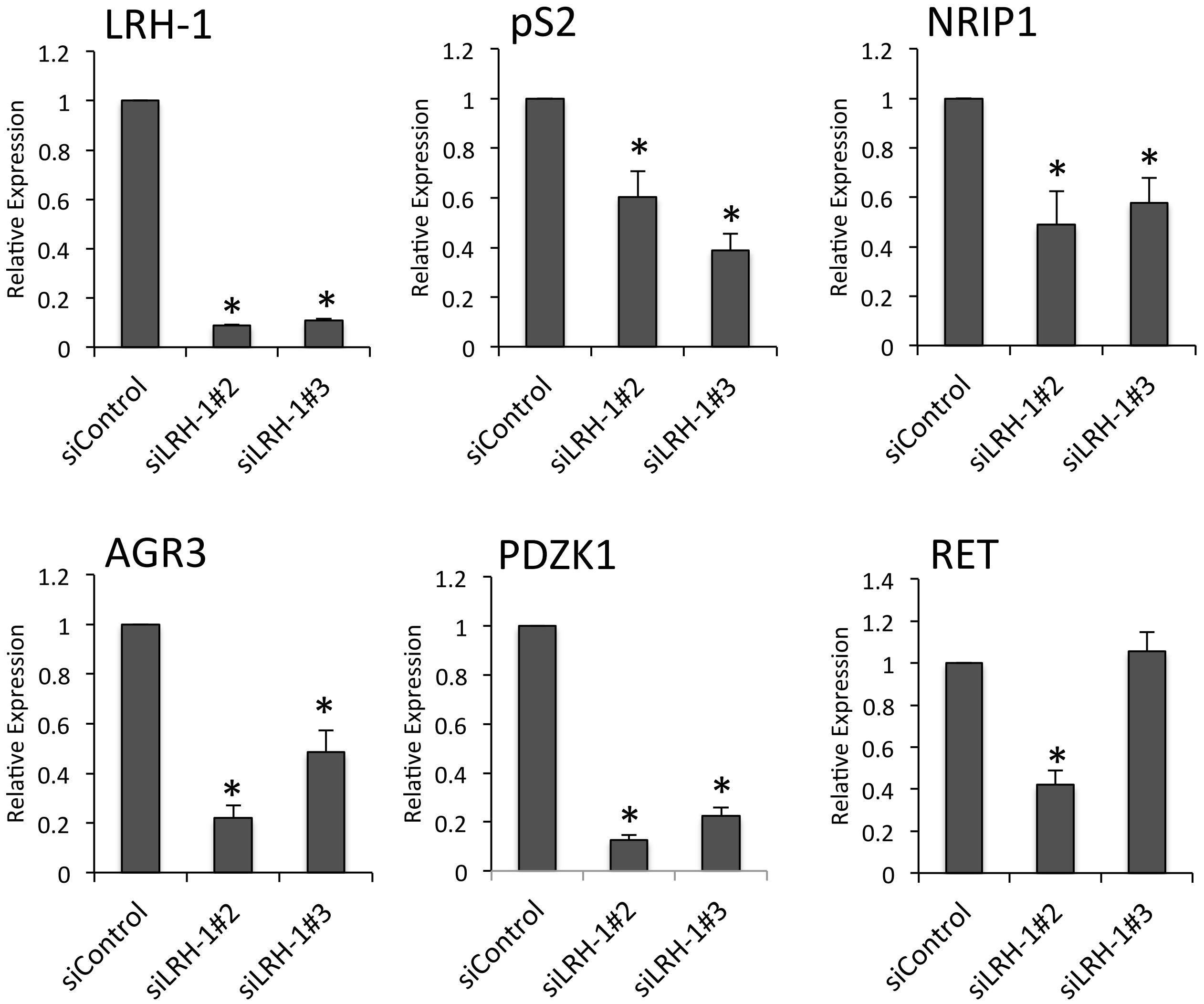

Supplementary Figure 8. Quantitative RT-PCR was performed using RNA prepared from MCF-7 cells were transfected with two siRNAs targeting LRH-1. Expression is shown relative to siControl, following normalization against GAPDH expression (n=3, error bars = SEM, * = $p<0.05$ for siLRH-1 compared with siControl transfections).

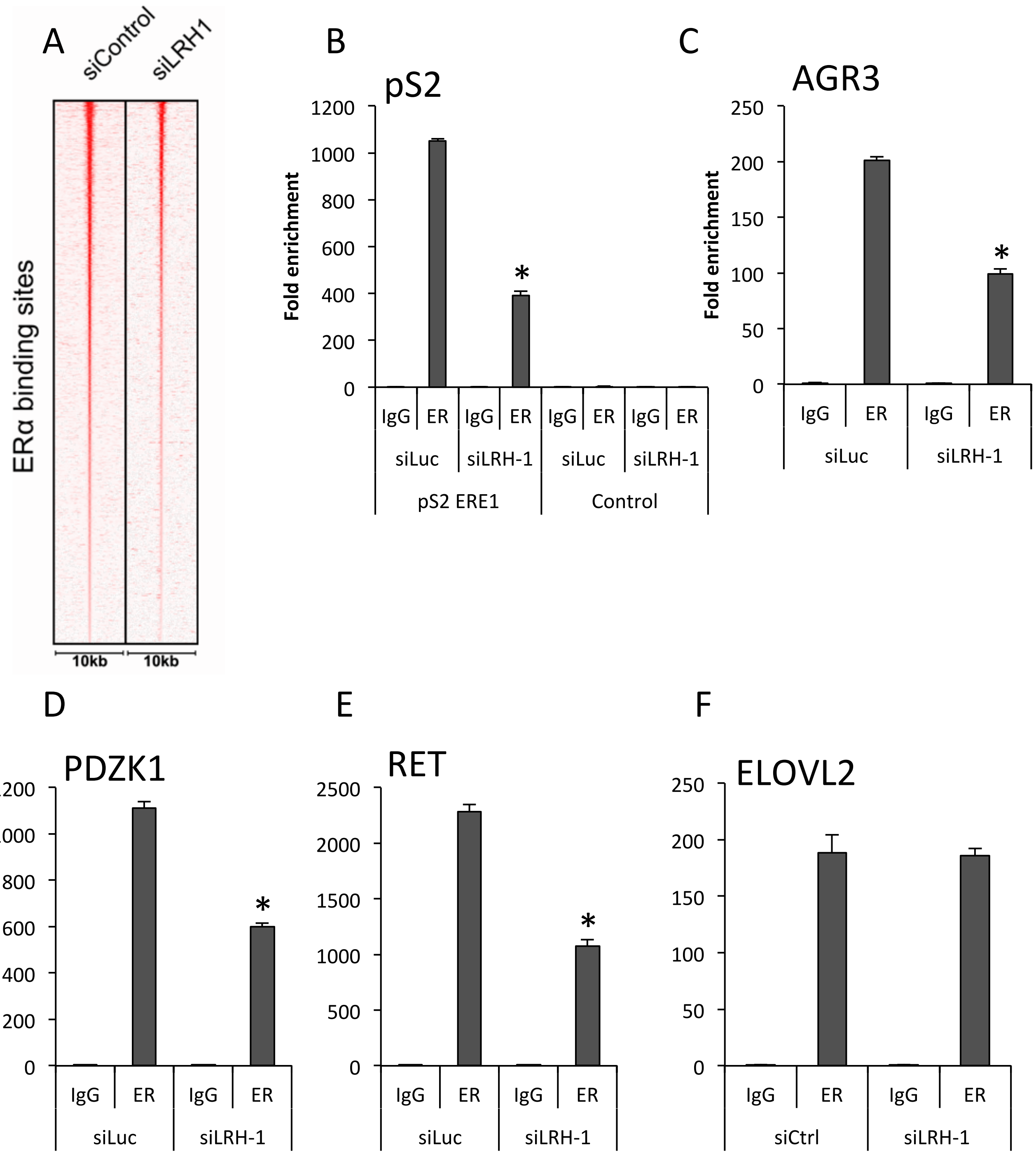

Supplementary Figure 9. (A) ChIP-seq for ERα was performed following MCF-7 cell transfection with siControl or siLRH-1 (n=3). Heatmap depicting all the ERα binding is shown, with a window of 10 kb around the binding sites. (B-F) ChIP for ERα using primers flanking the ERα binding regions highlighted in Figure 4D is shown (n=3; error bars = SEM). * represents significant ($p<0.01$) differences between ER binding for siLRH-1, compared with siLuc transfections.

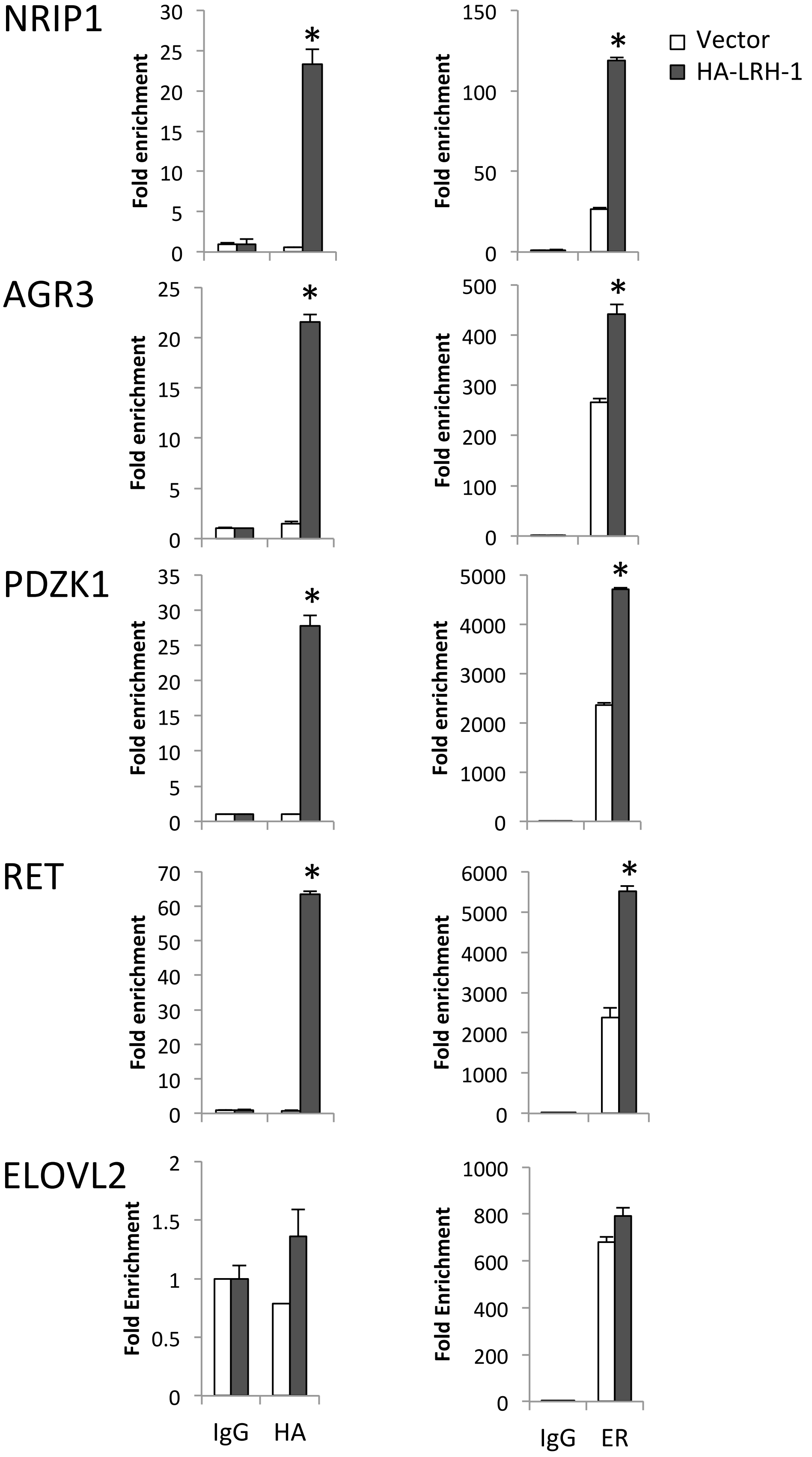

Supplementary Figure 10. LRH-1 ChIP using MCF-7 lysates is shown. ERα ChIP with these lysates is shown in Figure 4E. * denotes significant ($p<0.01$) differences between vector control and LRH-1 transfected samples (n=3).

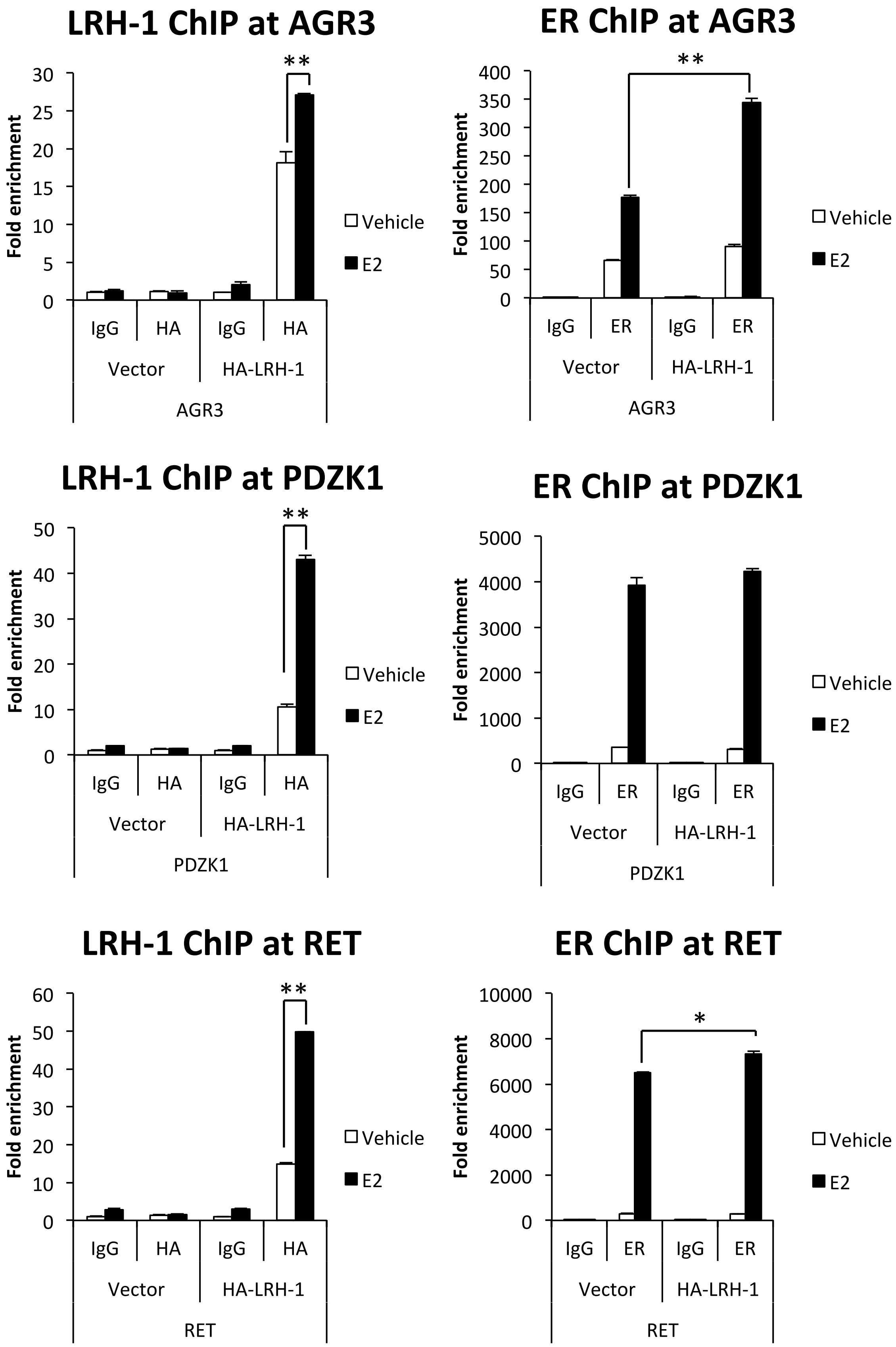

Supplementary Figure 11. Q-PCR was performed following ChIP as described in the legend to Figure 6. **p < 0.01, *p < 0.05.

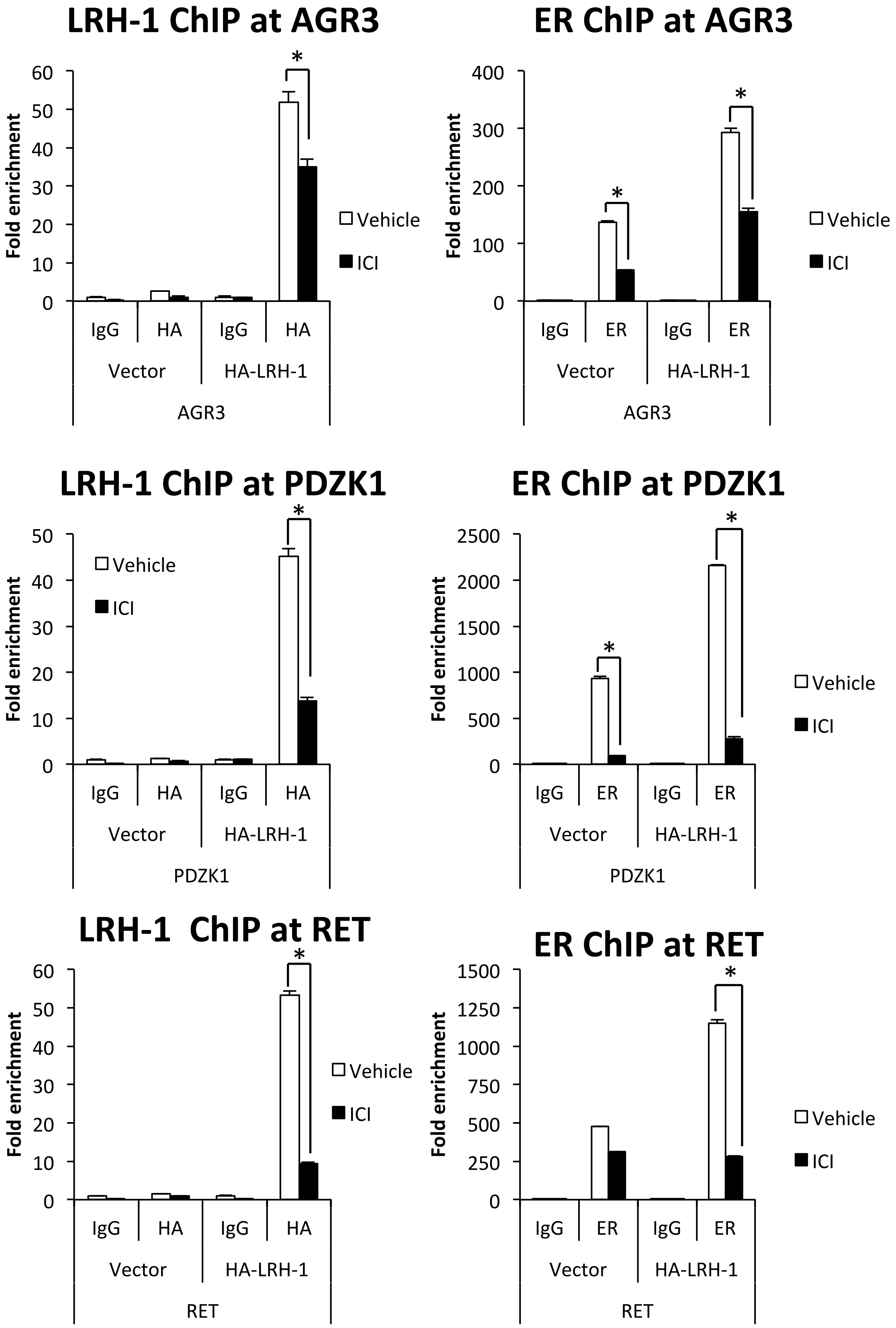

Supplementary Figure 12. Q-PCR was performed following ChIP as described in the legend to Figure 6. ICI = ICI182780. * $p < 0.01$.

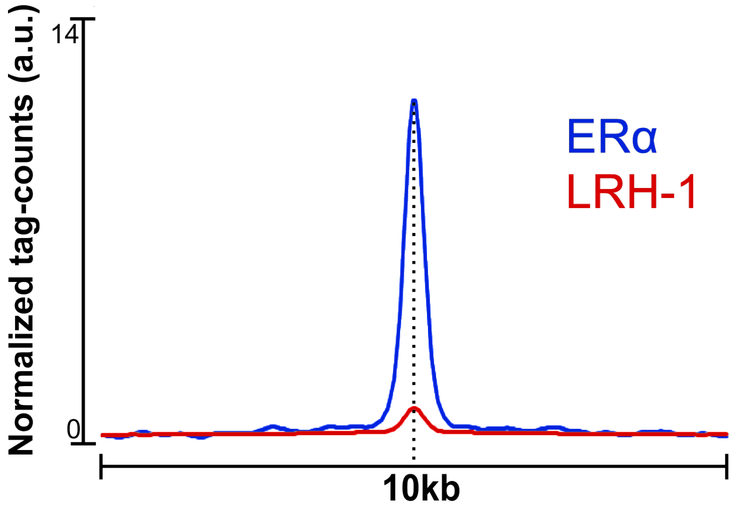

Supplementary Figure 13. 2D graph visualization of the normalized tag-count (a.u.) of ERα (blue) and LRH-1 (red) CHIP-seq signal at chromatin binding sites, selectively ERα unique sites. Although the peak calling algorithm does not identify LRH-1 binding at these sites, low intensity signal was observed. Dotted line indicated the centre of the peak region.

Supplementary Information – Lai et al

Sequences of oligonucleotides used for the Electrophoretic Mobility Shift Assay (EMSA)

**pS2 ERE:**	5′-gatcCCCT**GCAAGGTCACGGTGGCCA**CCCCGTGA-3′<br>5′-gatcTCACGGGG**TGGCCACCGTGACCTTGC**AGGG-3′
**pS2 EREm1:**	5′-gatcCCCT**GgtAGGTCACGGTGGCCA**CCCCGTGA-3′<br>5′-gatcTCACGGGG**TGGCCACCGTGACCTacC**AGGG-3′
**pS2 EREm2:**	5′-gatcCCCT**GCAAccTCACGGTGGCC**ACCCCGTGA-3′<br>5′-gatcTCACGGGG**TGGCCACCGTGAggTTGC**AGGG-3′
**pS2 EREm3:**	5′-gatcCCCT**GCAAGGTCACGGTGGggA**CCCCGTGA-3′<br>5′-gatcTCACGGGG**TccCCACCGTGACCTTGC**AGGG-3′
**pS2 EREm4:**	5′-gatcCCCT**GCAAccTCACGGTGGgg**ACCCCGTGA-3′<br>5′-gatcTCACGGGG**TccCCACCGTGAggTTGC**AGGG-3′
**NRIP1 ERE:**	5'-CAGC**TCAAGGTCACACTAACCC**GCCCT-3'<br>5'-AGGGC**GGGTTAGTGTGACCTTGA**GCTG-3'
**NRIP1 EREm1:**	5'-CAGC**TgtAGGTCACACTAACCC**GCCCT-3'<br>5'-AGGGC**GGGTTAGTGTGACCTacA**GCTG-3'
**NRIP1 EREm1:**	5'-CAGC**TCAAttTCACACTAACCC**GCCCT-3'<br>5'-AGGGC**GGGTTAGTGTGAaaTTGA**GCTG-3'
**vit ERE:**	5′-gatcCAAAGTC**AGGTCACAGTGACCT**GATCAAAGA-3′<br>5′-gatcTCTTTGATC**AGGTCACTGTGACCT**GACTTTG-3′
**vit EREm1:**	5′-gatcCAAAGTC**AccTCACAGTGACCT**GATCAAAGA-3′<br>5′-gatcTCTTTGATC**AGGTCACTGTGAggT**GACTTTG-3′
**vit EREm2:**	5′-gatcCAAAGca**AGGTCACAGTGACCT**GATCAAAGA-3′<br>5′-gatcTCTTTGATC**AGGTCACTGTGACCT**tgCTTTG-3′
**vit EREm3:**	5′-gatcCAAAtca**AGGTCACAGTGACCT**GATCAAAGA-3′<br>5′-gatcTCTTTGATC**AGGTCACTGTGACCT**tgaTTTG-3′
**AFP:**	5′-CTATCTTATGT**TCAAGGACA**AAGACCACTTC-3′<br>5′-GAAGTGGTCTT**TGTCCTTGA**ACATAAGATAG-3′
**AFPm1:**	5′-CTATCTTATGT**TgtAGGACA**AAGACCACTTC-3′<br>5′-GAAGTGGTCTT**TGTCCTacA**ACATAAGATAG-3′

Sequences of Oligonucleotides used to generate pS2-luc mutants by Site-Directed Mutagenesis

pS2-EREm1-luc	5′-TCCCTTCCCCCTGGgtAGGTCACGGTGGCC-3′
pS2-EREm2-luc	5′-TCCCTTCCCCCTGCAAccTCACGGTGGCC-3′
pS2-EREm3-luc	5′-AAGGTCACGGTGGttACCCCGTGAGCCA-3′

Mutagenesis of pS2-EREm2-luc was performed to generate pS2-EREm4-luc using an oligonucleotide

pS2-EREm4-luc	5'-AAccTCACGGTGGttACCCCGTGAGCCA-3′

Sequences of PCR Primers used for Chromatin immunoprecipitation (ChIP):

pS2 ERE:	5′-TATGAATCACTTCTGCAGTGAG-3′
	5′-GAGCGTTAGATAACATTTGCC-3′
Control:	5′-GTGATTCTCCTGACTTAACC-3′
	5′-TGGCGCAGTGGCTCACGCTG-3′
NRIP1:	5′-CTGCCCCATTCTTTGTGATT-3′
	5′-TAGCAGGATCTGTGCCAGTG-3′
AGR3:	5′-TTTGAGCTGCCCTGTGCTAA-3′
	5′-GCTGATGGCATTTTGGCCTC-3′
PDZK1	5′-AGGCCCAGCAAAGACAAATG-3′
	5′-AAACCACAGGCTGAGGACTG-3′
RET	5′- CTGAGGGCGCAGAGATACAG-3′
	5′- TGACTCAGCCAGTCTCACCT-3′

Real-time RT-PCR Assays

Gene Name	ABI Assay Catalogue Number
LRH-1 (NR5A2)	Hs00892377_m1
ERα (ESR1)	Hs00174860_m1
pS2 (TFF1)	Hs00170216_m1
GREB1	Hs00536409_m1
NRIP1	Hs00942766_m1
Cathepsin D (CTSD)	Hs00157201_m1
AGR3	Hs00411268_m1
PDZK1	Hs00275727_m1
RET	Hs01120030_m1
GAPDH	Hs99999905_m1

Antibodies

	Applications	Catalogue Number	Supplier
ERα (ESR1)	ChIP	sc-543	Santa Cruz
HA	ChIP	04-902	Millipore
GATA3	ChIP, Immunoblotting	sc-268	Santa Cruz
FOXA1	ChIP, Immunoblotting	ab5089, ab23738	Abcam
RNA polymerase II (PolII)	ChIP, Immunoblotting	05-623	Millipore
CBP	ChIP, Immunoblotting	sc-369	Santa Cruz
p300	ChIP, Immunoblotting	sc-585	Santa Cruz
AIB1	ChIP, Immunoblotting	611105	BD Biosciences
Ac-H3K9	ChIP	ab4441	Abcam
Ac-H3K27	ChIP	ab4729	Abcam
Histone H3	ChIP	ab1791	Abcam
IgG	ChIP	#2729	Cell Signalling
LRH-1	Immunoblotting	PP-H2325-00	Perseus Proteomics
ERα (ESR1)	Immunoblotting	NCL-ER-6F11	Novacastra Laboratories
HA	Immunoblotting	sc-7392	Santa Cruz
TFF1 (pS2)	Immunoblotting	sc-28925	Santa Cruz
ß-actin	Immunoblotting	ab6276	Abcam
